# Donor KIR2DS1 reduces the risk of transplant related mortality in HLA-C2 positive young recipients with hematological malignancies treated by myeloablative conditioning

**DOI:** 10.1371/journal.pone.0218945

**Published:** 2019-06-25

**Authors:** Attila Tordai, Andras Bors, Katalin Piroska Kiss, Katalin Balassa, Hajnalka Andrikovics, Arpad Batai, Aniko Szilvasi, Katalin Rajczy, Dora Inotai, Eva Torbagyi, Lilla Lengyel, Aniko Barta, Peter Remenyi, Tamas Masszi

**Affiliations:** 1 Laboratory of Molecular Diagnostics, Hungarian National Blood Transfusion Service, Budapest, Hungary; 2 Department of Pathophysiology, Semmelweis University, Budapest, Hungary; 3 Laboratory of Molecular Diagnostics, South-Pest Central Hospital–National Institute of Haematology and Infectology, Budapest, Hungary; 4 Department of Haematology and Stem Cell Transplantation, South-Pest Central Hospital–National Institute of Haematology and Infectology, Budapest, Hungary; 5 Transplantation Immunogenetics Laboratory, Hungarian National Blood Transfusion Service, Budapest, Hungary; 6 Hungarian Stem Cell Donor Registry, Hungarian National Blood Transfusion Service, Budapest, Hungary; 7 3^rd^ Department of Internal Medicine, Semmelweis University, Budapest, Hungary; University of Kentucky, UNITED STATES

## Abstract

**Background:**

Recognition of HLA-C2 group alleles on recipient cells by activating killer immunoglobulin like receptors, KIR2DS1 on donor natural killer cells may lead to increased graft-versus-leukemia effect or immunomodulation in patients treated by allogeneic hematopoietic stem cell transplantation (allo-HSCT) influencing disease free and overall survival (OS).

**Objective:**

In the present study, 314 consecutive, allo-HSCT recipient and donor pairs were included with retrospective donor KIR-genotyping and clinical parameters analyzes.

**Results:**

After a median follow-up of 23.6 months, recipients with HLA-C2 group allele (rC2) showed improved (p = 0.046) OS if transplanted with KIR2DS1 positive donors (d2DS1) compared to those without one or both of this genetic attribute. Within the myeloablative conditioning (MAC) subgroup (n = 227), rC2 homozygous+d2DS1 patients (n = 14) showed a 5 years OS of 93% followed by rC2 heterozygous+d2DS1 patients (n = 48, 65%) compared to rC2 and/or d2DS1 negatives (47%, p = 0.018). Multivariate analyses indicated rC2+d2DS1 positivity as an independent predictor of OS (HR:0.47, 0.26–0.86, p = 0.014) besides donor type, presence of CMV-reactivation or chemoresistant disease. Among MAC-treated patients, the combined rC2+d2DS1 presence was associated with a markedly decreased cumulative incidence of transplant related mortality (p = 0.0045).

**Conclusion:**

The combination of rC2+d2DS1 may be a favorable genetic constellation in allo-HSCT with MAC potentially reducing transplant related mortality.

## Introduction

In adults, allogeneic hematopoietic transplantation (allo-HSCT) is the exclusive curative therapeutic option in several hematologic malignancies primarily acute leukemias. However, this intervention has severe and frequent complications such as relapse and transplant related mortality (TRM) caused primarily by acute or chronic graft versus host disease (aGvHD or cGVHD) [1–3] due to interactions between the multitude of genetic and acquired factors of recipients and donors [4–6]. Among these factors, recent attention has also turned towards killer immunoglobulin like receptors (KIR) on natural killer (NK) cells [7–9]. Cell surface KIR-expression is stochastic with individual cells expressing different numbers and types of receptor protein in a probabilistic manner [7]. This makes the genetic testing approach the single current feasible technique in cohort-studies to estimate KIR-expression. NK-cells are known to play an important role in anti-tumor immune response with a complex interplay between activating and inhibitory KIR-s (aKIR and iKIR). In addition, NK-cells have also been implicated in post-transplant immunomodulation potentially decreasing TRM and improving infection control [9,10]. KIR ligands are specific regions of class I human leukocyte antigen (HLA) molecules on target cells. For one of the most studied aKIR, KIR2DS1, the ligand requirement is a lysine amino acid at position 80 (Lys80) of the HLA-C alpha chain. Due to dimorphism, individuals may be homozygous HLA-C2 group allele carriers (C2/C2, Lys80/Lys80), C1/C2 heterozygotes (Asn80/Lys80) or C1/C1 homozygotes (Asn80/Asn80) [11]. Upon recognition of their ligand, aKIR-s may contribute to graft versus leukemia (GvL) effect or exert further immunomodulation. In contrast, ligation of iKIR by their ligands may lead to decreased NK-cell activity (i.e. tolerance) while in the absence of the appropriate ligand, more pronounced NK allo-reactivity may occur [12].

The most extensively studied model of NK-cell role in HSCT is the “missing self” model in which iKIR-s play a role by not transmitting inhibitory signals due to missing ligand thus augmenting GvL activity [13]. In matched transplantations, another approach has been the isolated study of the presence or absence of individual aKIR-s [14,15] or iKIR-s [16,17] in combination with their ligands. In these approaches, the main outcome parameter has usually been relapse free survival with an observed favorable effect of donor KIR2DS1 and recipient HLA-C1 improving GvL activity [14]. Additional studies were performed focusing on the entire KIR haplotype comparing the activation dominant cen-B haplotype with cen-A (higher prevalence of iKIR-s) and confirming the beneficial effect of the cen-B against relapse [18–21]. NK-cell licensing has recently been studied emphasizing the importance of prior coexistence of aKIR-s and iKIR-s with their respective ligand during NK-cell maturation in the donor [9,14].

Due to the complexity of allo-HSCT as well as the numerous cell types, receptors, ligands and soluble factors involved in immunological reconstitution, to date no generally accepted agreement has been reached regarding the use of KIR-genotyping information during donor selection. Thus, the aim of the current study was to focus on the joint role of recipient HLA-C2 group allele and donor KIR2DS1 in allo-HSCT outcome with special emphasis on the interaction of these genetic attributes with those of known factors of this complex intervention.

## Materials and methods

### Patients

This retrospective study included 314 consecutive, Caucasian, adult patients (median age: 41 years, range: 19–73), who underwent first allo-HSCT between January 2007 and December 2013 at our single center in Hungary, for acute myeloid leukemia (AML, n = 159), acute lymphoblastic leukemia (ALL, n = 73), myelodysplastic syndrome (MDS, n = 35), chronic myeloid leukemia (CML, n = 24), myeloproliferative neoplasm (MPN, n = 23), with the latter 3 groups collectively considered as: MDS+CML+MPN = 82. Among acute leukemia patients (n = 232), 190 (81%) were transplanted in complete remission while in 42 cases (19%) this was not achieved prior to transplantation (chemo-resistant cases). The sibling/unrelated donor (UD) composition of our cohort was close to equal (153/161). Except for 9 cases (8 bone marrow and 1 cord blood), peripheral blood was the graft source. Considering 4 HLA loci on the antigenic level, a total of 46 patients (15%) received a graft from a donor with at least one mismatch (4 sibling donors). Of the 46 mismatched pairs, HLA-A was affected in 13, HLA-B in 2, while HLA-C in 31 cases. HLA-DRB1 mismatch at the antigen level did not occur. Of the 31 HLA-C mismatched cases, 11 donors carried HLA-C1/C1 types out of whom 5 cases were identified in which the recipient was positive for one or two HLA-C2 group alleles and, among these 5 cases, only a single donor was positive for KIR2DS1. Mismatched transplants were not excluded from the analyses, but multivariate models were systematically adjusted for mismatching. Myeloablative conditioning (MAC) was applied in the majority (n = 227, 72%) of cases with the predominance of total body irradiation+cyclophosphamide (n = 147, 64% of all MAC) followed by busulfan (n = 52, 23%) and total body irradiation+etoposide/melphalan (n = 28, 13%). In 87 cases (28%), reduced intensity conditioning (RIC) was performed. Post-transplantation immunosuppressive regimens (cyclosporine, tacrolimus, sirolimus) for GvHD prevention were employed depending on clinical conditions. Acute GvHD was defined and graded according to consensus criteria [22], cGVHD was classified as absent, limited or extended [23]. Infection as cause of death was determined in the absence of relapse and in the presence of microbiological evidence of bacterial, viral or fungal microorganisms.

The study was approved by the Hungarian National Ethics Committee and was in accordance of the Declaration of Helsinki. All patients provided written informed consent.

### HLA- and KIR-typing

Whole genomic DNA was isolated from peripheral blood or bone marrow by standard commercial kits. Low or medium resolution HLA-A, -B, -C and -DRB1 typing for recipients and donors in the sibling donor setting were performed by polymerase chain reaction (PCR) with sequence specific primers (SSP, Olerup, Stockholm, Sweden) or sequence specific oligonucleotide probes (SSO, One Lambda, Los Angeles, CA, USA). For unrelated HSCT, high resolution HLA-typing was performed for HLA-A, -B, -C, -DRB1, and -DQB1 by high resolution SSP (Olerup) or sequence based typing (SBT, Qiagen, ROSE, Valencia, CA, USA). HLA-C1/2 group allele assignment was based on the respective HLA-C types (asparagine at position 80 for C1, lysine for C2 group alleles). In the sibling donor subgroup (n = 153), HLA-C1/C2 calling by low or medium resolution typing was only ambiguous in a small number of cases (e.g. HLA-C*03, *07, *12, *15, *16) which were resolved by high resolution typing. Genotyping for the presence of KIR genes was performed by an allele specific multiplex PCR using archived DNA samples of HSCT donors [24]. Briefly, amplifications of 14 genes/variants with 31 primer pairs (oligonucleotide sequences were identical to those of Abalos et al. and are available upon request) were performed in 4 multiplex PCR reactions, followed by size separation on agarose gel-electrophoresis. Genotyping calls were made on the bases of amplicon presence or absence with the respective sizes.

### Statistical methods

Categorical variables were analyzed by the χ2 or the Fisher’s exact tests while continuous variables by the Mann-Whitney test. Overall survival (OS) was defined as the survival from the day of transplantation until death from any cause or last follow-up. At dates of second allo-HSCT, the respective patients were censored. OS data were analyzed by the log-rank test and Kaplan-Meier estimates were computed. Following stratification for the respective variable (e.g. donor type, conditioning, etc.) hazard ratio (HR) values were calculated along with 95% confidence intervals in univariate Cox proportional hazard models. In multivariate survival analyses, Cox models were adjusted for age, sex, donor type, HLA-matching, conditioning intensity (SPSS Statistics Software v.22). Event free survival (EFS) was defined as survival until relapse or death from any cause. EFS comparisons were made with Kaplan-Meier estimates and the log-rank test. These latter 2 analyses were performed with EZR (Easy R) [25]. Hazards of relapse and aGVHD were analyzed in multivariate competing risk (death as competing event) regression models. Transplant related mortality (TRM) was defined as death without relapse. TRM incidence rates were calculated in person-years and compared using exact confidence intervals with the Stata Program v.15.0 [26–28].

## Results

The genotype distribution of HLA-C group alleles among recipients were as follows: 118/314 (38%) C1/C1, 144/314 (46%) C1/C2 and 52/314 (16%) C2/C2. The activating KR2DS1 gene was present in 119/314 (38%) donors while 195 (62%) of them were negative for KR2DS1. The combined presence of donor KIR2DS1 (hereinafter termed as d2DS1) and its ligand, recipient HLA-C2 (hereinafter termed as rC2) was observed in 69/314 (22%) allo-HSCT pairs. Comparing the basic demographics, disease and intervention related characteristics as well as frequencies of key outcome parameters between the jointly positive rC2+d2DS1 (n = 69) subgroup and the rest of the cohort (n = 245), no significant differences were observed except for the age at transplantation (Table 1). Allo-HSCT patients with the combined rC2+d2DS1 genotype were significantly younger compared to pairs with other combinations (median ages 36 vs. 42 years, p = 0.028). To control for the observed age bias, we systematically included age in all subsequent statistical analyzes.

**Table 1:** Basic demographic, disease and intervention related characteristics as well as presence or absence of key outcome parameters for the entire cohort and for subgroups according to the simultaneous presence of recipient HLA-C2 group alleles (rC2) and donor KIR2DS1 (d2DS1) gene.

Characteristics	Total		rC2 and d2DS1 pos.*		rC2 and/or d2DS1 neg.**		
	n	%	n	%	n	%	p
Number of patients	314		69		245		
**Recipient age at TX**	** **	** **	** **	** **	** **	** **	**0.028**
Below median	157		43	62%	114	47%	
Above median	157		26	38%	131	53%	
**Gender**	** **	** **	** **	** **	** **	** **	0.89
Female	145	46%	31	45%	114	47%	
Male	169	54%	38	55%	131	53%	
**Diagnosis**	** **	** **	** **	** **	** **	** **	0.78
AML	159	51%	37	54%	122	50%	
ALL	73	23%	14	20%	59	24%	
MDS+CML+MPN	82	26%	18	26%	64	26%	
**Donor**	** **	** **	** **	** **	** **	** **	0.22
Sibling	153	49%	29	42%	124	51%	
Unrelated (UD)	161	51%	40	58%	121	49%	
**HLA-match 8/8 antigen level**	** **	** **	** **	** **	** **	** **	0.17
8/8	268	85%	55	80%	213	87%	
Mismatch	46	15%	14	20%	32	13%	
**Conditioning intensity**	** **	** **	** **	** **	** **	** **	0.56
MAC	227	72%	48	70%	179	73%	
RIC	87	28%	21	30%	66	27%	
**Acute GvHD**	** **	** **	** **	** **	** **	** **	0.87
No GvHD	128	41%	28	41%	100	41%	
Grade 1	87	28%	22	32%	65	27%	
Grade 2	59	19%	12	17%	47	19%	
Grade 3	26	8%	5	7%	21	9%	
Grade 4	14	4%	2	3%	12	5%	
**CMV reactivation/disease**	** **	** **	** **	** **	** **	** **	1.00
Yes	59	19%	13	19%	46	19%	
No	255	81%	56	81%	199	81%	
**Chemoresistant disease**	** **	** **	** **	** **	** **	** **	0.23
Yes	42	13%	6	9%	36	15%	
No	272	87%	63	91%	209	85%	
**Relapse/progression**	** **	** **	** **	** **	** **	** **	0.87
Yes	71	23%	16	23%	55	22%	
No	243	77%	53	77%	190	78%	

*rC2 positivity included both, patients (recipients) with HLA-C1/C2 and C2/C2 genotypes.

**This cohort contained rC2 pos. and d2DS1 neg. (n = 127), rC2 neg. and d2DS1 pos. (n = 50) and rC2 neg. and d2DS1 neg. (n = 68) transplant pairs.

p values below 0.05 are indicated with boldface character.

**Abbreviations**: AML = acute myeloid leukemia, ALL = acute lymphoblastic leukemia, CML = chronic myeloid leukemia, CMV = cytomegalovirus, d2DS1 = donor KIR2DS1 gene, GvHD = graft-versus-host disease, MAC = myeloablative conditioning, MDS = myelodysplastic syndrome, MPN = myeloproliferative neoplasm, rC2 = recipient HLA-C2 group allele, RIC = reduced intensity conditioning, TX = transplantation.

**Fig 1:**
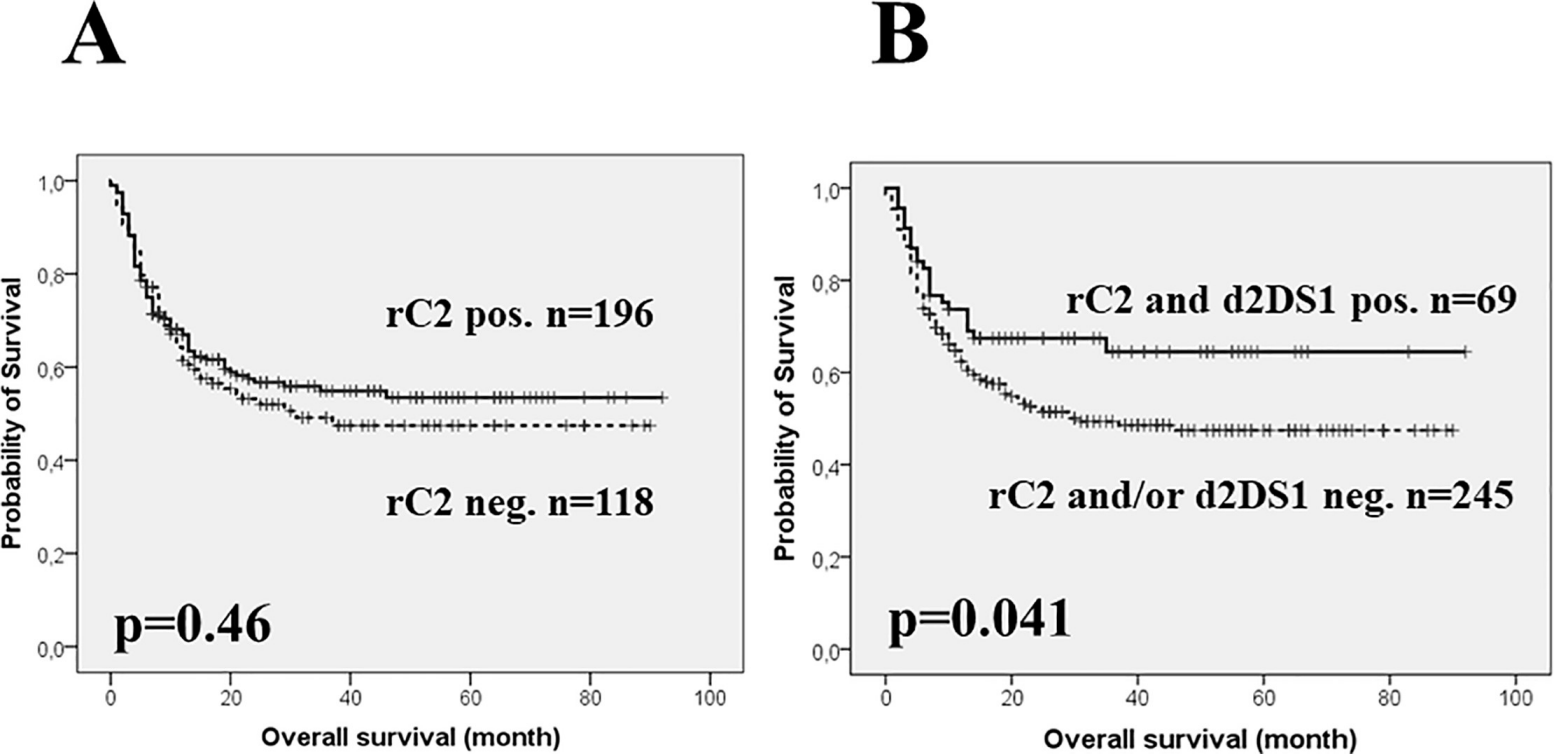
**Overall survival of the entire allo-HSCT cohort (n = 314) according to recipient HLA-C2 group allele positivity (panel A) and combined presence of recipient HLA-C2 + donor KIR2DS1 (panel B).** Survival analyses were performed by the Kaplan-Meier method and compared with the log rank test with probability (p) values and subgroup case numbers indicated. In panel A, solid line = recipients with 1 or 2 HLA-C2 group alleles (n = 196), dashed line = recipients with homozygous HLA-C1 group alleles (n = 118). In panel B, solid line = recipients with 1 or 2 HLA-C2 group alleles + donors with KIR2DS1 genotype (n = 69), dashed line = recipients with homozygous HLA-C1 group alleles and KIR2DS1 positive donor + recipients with 1 or 2 HLA-C2 group alleles and lack of KIR2DS1 in the donor (n = 127). Abbreviations: d2DS1 = donor KIR2DS1 gene, pos = positive, neg = negative, rC2 = recipient HLA-C2 group allele.

As an important outcome parameter, OS was compared by the Kaplan-Meier method in the entire cohort separately according to rC2 positivity and d2DS1 presence. OS for recipients with rC2 positivity (C1/C2 n = 144 and C2/C2 n = 52 combined) did not differ from those of rC2 negatives (Fig 1A, 5-year survival rates of 53% vs. 47%, p = 0.46). Transplantation of rC2 positive recipients with d2DS1 positive donors resulted in a significant improvement in OS compared to those pairs which were negative for either or both genetic factors (Fig 1B) with 5-years survival rates of 64% (± 6.2%) vs. 47% (± 3.6%, p = 0.041).

**Fig 2:**
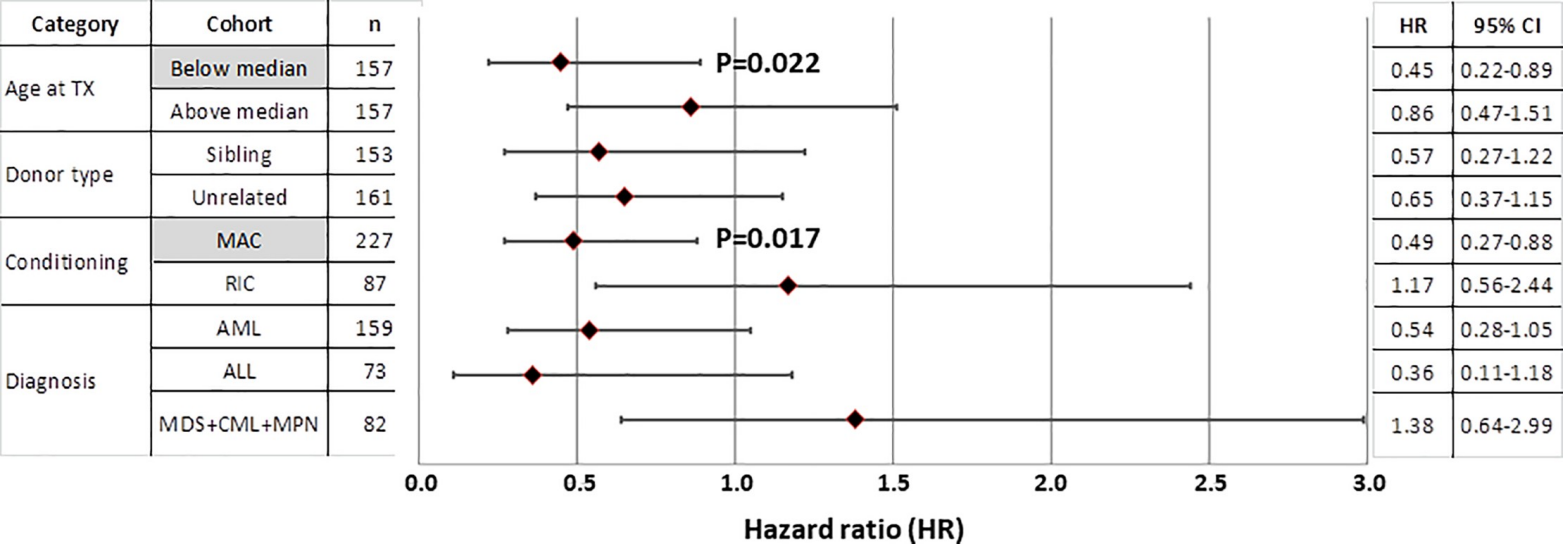
Effect of rC2+d2DS1 combination on overall survival (OS) as measured by multivariate Cox regression analyses in various subgroups. Multivariate Cox proportional hazard models were used calculating hazard ratio (HR) values along with 95% confidence intervals (95% CI) and p values (bold face character if > 0.05) after selecting the indicated subgroup using age, sex, donor type, conditioning and diagnosis as covariates in all subgroups. Cohort sizes (n) are indicated on the left, while HR values with 95% CI on the right. The presence of rC2+d2DS1 significantly improved OS in only 2 subgroups (grey filled cells), for which p values are indicated. Other p values were not significant (>0.05) and are not shown. **Abbreviations**: AML = acute myeloid leukemia, ALL = acute lymphoblastic leukemia, CI = confidence interval, CML = chronic myeloid leukemia, d2DS1 = donor KIR2DS1 gene, GvHD = graft-versus-host disease, HR = hazard ratio, MAC = myeloablative conditioning, MDS = myelodysplastic syndrome, MPN = myeloproliferative neoplasm, rC2 = recipient HLA-C2 group allele, RIC = reduced intensity conditioning, TX = transplantation.

In order to search for attributes influencing the favorable OS-effect, a series of multivariate analyses were performed in Cox regression models calculating HR values for OS in various subgroups (Fig 2). Dichotomous subgrouping of our cohort by age at transplantation indicated that OS was favorably affected by the combined presence of rC2+d2DS1 among younger patients (HR = 0.45, p = 0.022) while such an effect was not observed in the older half of our cohort. The most profound distinction was found with respect to the conditioning regimen. The favorable effect of rC2+d2DS1 on OS was significant in the MAC-treated subgroup (HR = 0.49, p = 0.017) while OS was unaffected by the genetic combination among RIC-treated recipients.

**Fig 3:**
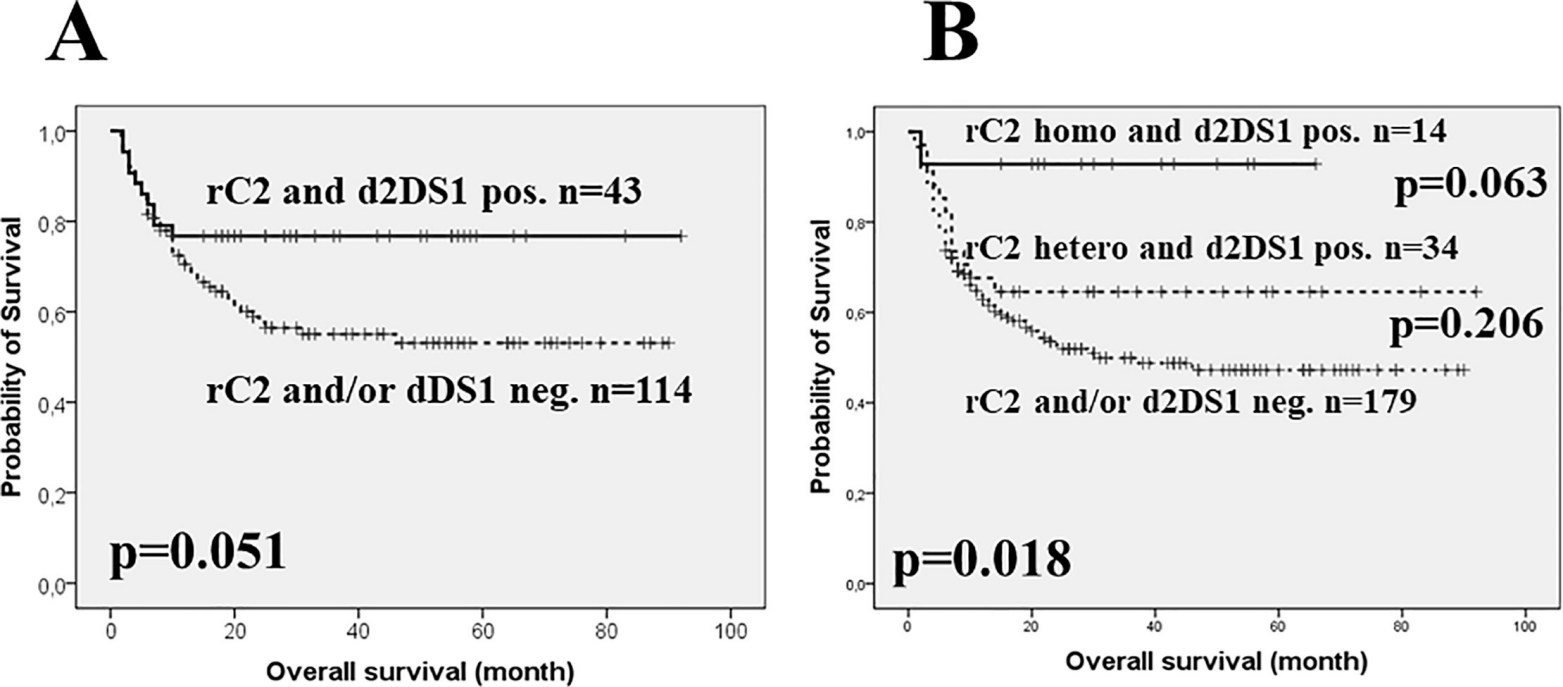
**Overall survival of the subgroup with age at TX below median (n = 157, panel A) and with myeloablative conditioning (n = 227, panel B) according to combined genotypes of recipient HLA-C2 and donor KIR2DS1.** Survival analyses were performed by the Kaplan-Meier method and compared with the log rank test with probability (p) values and subgroup case numbers indicated. In panel B, p values indicating dichotomous comparisons are shown between the respective curves, while the global p value for the overall comparison is shown in the left bottom corner of the graph. In panel A, among younger patients, solid line = recipients with 1 or 2 HLA-C2 group alleles + donors with KIR2DS1 genotype (n = 43), dashed line = recipients with homozygous HLA-C1 group alleles and KIR2DS1 positive donor + recipients with 1 or 2 HLA-C2 group alleles and lack of KIR2DS1 in the donor (n = 114). In panel B, solid line = recipients with homozygous HLA-C2 group alleles + donors with KIR2DS1 genotype (n = 14), dashed line = recipients with heterozygous HLA-C1/C2 group alleles + donors with KIR2DS1 genotype (n = 34) and dotted line = recipients with homozygous HLA-C1 group alleles and KIR2DS1 positive donor + recipients with 1 or 2 HLA-C2 group alleles and lack of KIR2DS1 in the donor (n = 179). **Abbreviations**: d2DS1 = donor KIR2DS1 gene, pos. = positive, neg. = negative, rC2 = recipient HLA-C2 group allele.

Upon further Kaplan-Meier survival analyses in selected subgroups, among patients younger than the median of the entire cohort (n = 157), HLA-C2 group allele carrier recipients transplanted with KIR2DS1 positive donors (n = 43, 5-years survival rates of 77% ± 6.4%,) showed a markedly improved OS compared to those negative for one or both genetic attributes (n = 114, 5-years survival rates of 53% ± 5.2%, p = 0.051, Fig 3A). Within this younger subgroup, the distribution of transplant type was well balanced with 76/157 (48%) sibling and 81/157 (52%) UD transplants.

Among MAC-treated patients (n = 227), the similar dichotomous comparison resulted in a significant (p = 0.015) benefit for rC2+d2DS1 pairs (not shown). As shown on Fig 3B, further stratification of the rC2+d2DS1 subgroup according to HLA-C2 group allele status indicated an overall significant (p = 0.018) dose-effect of the KIR2DS1 ligand with the strongest OS-improvement among C2/C2 homozygous recipients (n = 14, 5-years OS of 93% ± 6.9%) followed by C1/C2 heterozygous patients (n = 34, 5-years survival rates of 65% ± 8.2%) compared to those negative for one or both genetic attributes (n = 179, 5-years OS of 47% ± 4.3%). The rC2 homozygous group (n = 14) with the best OS consisted of 6 sibling and 8 UD transplants. In the latter cases, 4 donors were identically HLA-C2 homozygous while 4 donors were HLA C1/C2 heterozygous. The similar distribution in the rC1/C2 heterozygous subgroup showed the following ratios: 12/34 (35%) sibling and 22/34 (65%) UD with 1/22 HLA-C2 homozygous donor and 21/22 HLA C1/C2 heterozygous donors.

To examine the interaction of the multitude of factors on OS among MAC-treated patients (n = 227), a Cox proportional hazard model was used in a multivariate setting containing all the indicated factors as covariates (Table 2). This analysis indicated that OS was independently affected by donor type (with sibling being favorable, p = 0.038), by the presence of cytomegalovirus (CMV) reactivation/infection (with reactivation being adverse, p = 0.022), by the presence of chemo-resistant disease (p<0.001) and by rC2+d2DS1 positivity (p = 0.014) considering age, sex, diagnosis, donor type and HLA-matching as covariates. In this comparison, rC2+d2DS1 positivity was an independent, favorable genetic factor for OS with a HR value of 0.47 (95% CI = 0.26–0.86).

**Table 2:** Multivariate analyses of various factors as covariates in a Cox proportional hazard model among MAC-treated patients (n = 227).

Covariate	HR	95% CI	p
Age at TX	1.01	0.99–1.03	0.115
Sex	1.21	0.81–1.81	0.358
Diagnosis (3 types)*	0.91	0.74–1.10	0.320
Donor type	1.63	1.03–2.59	**0.038**
HLA-matching	1.11	0.64–1.92	0.718
CMV reactivation/ disease	1.73	1.08–2.78	**0.022**
Chemoresistant disease	2.77	1.68–4.60	**<0.001**
rC2+dDS1 positivity	0.47	0.26–0.86	**0.014**

In this model, hazard ratio (HR) values for OS were calculated along with 95% confidence intervals (95% CI) and p values (bold face character if > 0.05) for all covariates by the Stata program.

*As above, the diagnosis was coded as AML, ALL and MDS+CML+MPN combined.

**Abbreviations**: CI = confidence interval, CML = chronic myeloid leukemia, CMV = cytomegalovirus, d2DS1 = donor KIR2DS1 gene, GvHD = graft-versus-host disease, HR = hazard ratio, MAC = myeloablative conditioning, rC2 = recipient HLA-C2 group allele.

**Fig 4:**
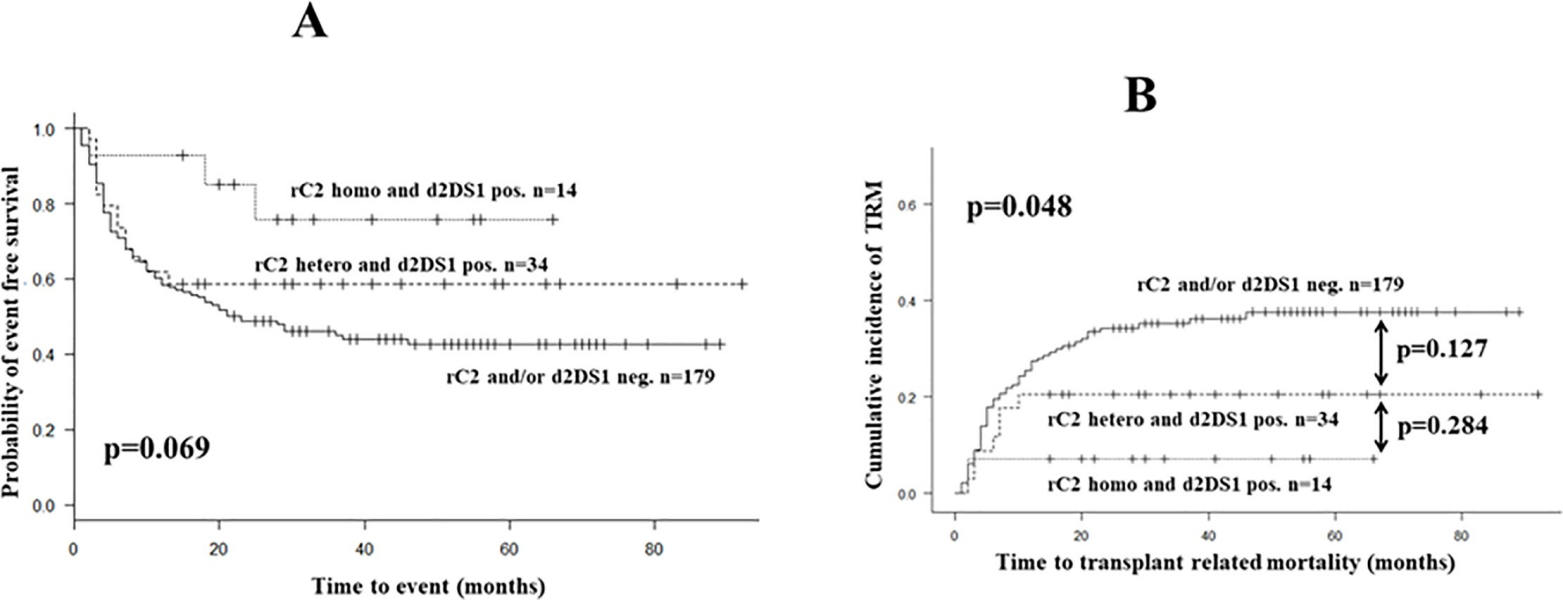
**Event free survival (EFS, panel A) and cumulative incidence of transplant related mortality (TRM, panel B) among TX-patients treated with MAC (n = 227) according to 3 possible combinations of combined genotypes of recipient HLA-C2 and donor KIR2DS1.** Event was defined as relapse or deaths from any cause, whichever came first. TRM was defined as death in the absence of relapse. Statistical comparisons were performed by the EZR program using Kaplan-Meier estimates and the log rank test for global comparison (panel A) and using Gray’s test for global (p = 0.048) and dichotomous comparisons (panel B). **Abbreviations**: d2DS1 = donor KIR2DS1 gene, pos. = positive, neg. = negative, rC2 = recipient.

Next, event free survival (EFS) was analyzed as an alternative outcome parameter and, a trend was observed towards improved EFS in the presence of rC2+d2DS1 combination with the highest EFS probability for HLA-C2 homozygous recipients with KIR2DS1 positive donors (n = 14, Fig 4A). The dichotomous comparison, i.e. rC2+d2DS1 positive patients combined (n = 48) vs. rC2 and/or d2DS1 negatives gave a lower and barely significant p value of 0.046 (not shown).

To dissect the two major factors behind the observed improved OS and EFS namely relapse and transplant-related mortality (TRM), we separately analyzed the cumulative incidence of relapse with death as competing event. To our surprise, relapse rates showed overlapping incidence curves upon comparing the rC2+d2DS1 positive (n = 48) vs. negative (n = 179) patient subgroups (not shown). In contrast, TRM showed decreased cumulative incidence in the presence of the specific KIR-related genetic constellation in both dichotomous (p = 0.021, not shown) comparisons and among recipient subgroups according to HLA-C2 homo- or heterozygosity (Fig 4B). with a significant (p = 0.048) global comparison.

To further substantiate or exclude the potential role of key factors for the observed OS improvement, hazard ratio (HR) values were separately calculated for cumulative incidences of relapse and aGVHD respectively using death as competing risk in a multivariate model among MAC-treated patients (n = 227). The presence of the rC2+d2DS1 combination did not exert a significant effect on either the cumulative incidence of relapse (p = 0.921) or on that of aGVHD (p = 0.535). In contrast, as expected, the presence of chemo-resistant disease adversely influenced relapse incidence (p<0.001) and transplantation with an unrelated donor adversely influenced aGVHD incidence (p<0.001). Interestingly, the presence of CMV reactivation/infection had a favorable effect on relapse incidence (p = 0.035, Table 3).

**Table 3:** Estimating the effect of various covariates on the hazard of relapse and acute GVHD respectively among MAC-treated patients (n = 227) in 2 separate multivariate competing risk regression models.

	Relapse		aGVHD grades 2 to 4	
Covariate	HR	p	HR	p
Age at TX	0.98	0.957	0.83	0.535
Sex	1.28	0.466	1.03	0.880
Diagnosis: AML vs. ALL	1.09	0.814	1.13	0.648
Diagnosis: MDS+CML+MPN vs. ALL	0.72	0.600	2.02	0.074
Donor type	1.36	0.396	**2.66**	**<0.001**
HLA-matching	1.43	0.38	0.87	0.652
CMV reactivation/ disease	**0.27**	**0.035**	1.59	0.103
Chemoresistant disease	**5.26**	**<0.001**	0.93	0.844
rC2+dDS1 positivity	0.96	0.921	0.82	0.535

In the models, hazard ratio (HR) values for relapse and aGvHD grades 2 to 4 combined were separately calculated along with 95% confidence intervals (not shown) and p values for all covariates by the Stata program. Among chemo-resistant patients (n = 42), relapse was coded at the time leukemia appearance upon bone marrow histology (n = 21) while in the other half of this subgroup, no evidence of leukemia was found during the entire follow-up. Bold face character indicates significance (<0.05).

**Abbreviations**: HLA-C1/C1: recipient homozygous for the HLA-C1 group alleles, HLA-C1/C2: recipient heterozygous for HLA-C2 group alleles, HLA-C2/C2: recipient homozygous for the HLA-C2 group alleles, aGvHD = acute graft-versus-host disease.

Next, we compared incidence rates of TRM within the MAC-treated subgroup with a total of 69/227 (30.4%) patients who died without relapse. The representation of rC2+dDS1 positive patients in the TRM-positive subgroup (8/69 = 11.6%) was significantly lower compared to that among TRM-negative patients (40/118 = 25.3%, p = 0.021, not shown). Furthermore, as shown in Table 4, among the 69 TRM-positive patients, the 8 patients positive for the rC2+dDS1 combination displayed a markedly lower TRM incidence rate of 0.066 person years compared to the 61 patients negative for the genetic combination with a TRM incidence rate of 0.174 person years (p = 0.0045).

**Table 4:** Comparison of transplant related mortality (TRM) incidence rates among MAC-treated patients (n = 227) according to the presence or absence of rC2+dDS1 positivity.

Category	Total, n	rC2+dDS1 positive	rC2 and/or dDS1 negative
TRM events, n	69	8	61
Time to OS, months	5658	1453	4205
TRM incidence rate, person years	—	0.066	0.174
Incidence rate ratio (95% confidence interval), exact p:		0.379 (0.156–0.796), **p = 0.0045**	

Overall cumulative incidence rates in person years were calculated by a multiplication of 12. Incidence rate ratio (0.066/0.174), exact 95% confidence intervals and p value were calculated by the Stata program. Bold face character indicates significance (<0.05).

**Abbreviations**: d2DS1 = donor KIR2DS1 gene, OS = overall survival, rC2 = recipient HLA-C2 group allele, TRM = transplant related mortality.

TRM may be a result of different complications such as aGVHD, cGVHD, infection or the combination of these. With respect to aGVHD grades 3/4, within the rC2+dDS1 positive subgroup (n = 8), a single patient (12.5%) suffered from aGVHD 3/4 while in the comparator group of 61 patients, 24 (39%) patients were diagnosed with aGVHD 3/4 (p = 0.430). Positivity for cGVHD (limited or extended) was found among 3/8 (38%) rC2+dDS1 positive transplant pairs while this ratio was 31/61 (51%) in the rC2 and/or dDS1 negative subgroup (p = 0.71). For infection as cause of death, relevant data for 5 transplantation cases were missing allowing 64 analyzes within the TRM-affected subgroup. Among the 6 rC2+dDS1 positive cases, all (100%) were diagnosed with infection as cause of death while this ratio was 46/58 (79%) in the comparator group (p = 0.58). Thus, we were unable to unequivocally identify a single pathophysiological mechanism for the observed substantial improvement in TRM-incidence.

## Discussion

In some respects, the current study had a similar design to that of the outstanding work by Venstrom et al. [14] analyzing a large cohort (n = 1277) of AML patients treated with unrelated donor (UD) transplantation and focusing on relapse as an outcome parameter. Their major finding was a favorable effect of the combined presence of donor KIR2DS1 and recipient HLA-C1 group alleles (i.e. a situation resembling “missing self”) while among patients with one or two HLA-C2 group allele, relapse rate did not improve. In a similar approach, Nowak et al. [15] essentially confirmed the above observations in a smaller UD-cohort (n = 285) with donor KIR2DS1 and recipient HLA-C2 group alleles showing inferior progression free survival and time to progression. Interestingly and surprisingly, in our cohort, we observed favorable outcomes in cases with the latter genetic combination however, for another important outcome parameter namely OS. This puzzling difference may be explained by our focus on MAC conditioning and the practically equal presence of sibling and UD transplants in our cohort (n = 112+115) translating to an overall lower ratio of HLA-mismatched donor-recipient pairs. In fact, we only identified a single pair in which the KIR2DS1 positive donor NK cells encountered HLA-C2 positive recipient cells after transplantation corresponding to the classical setting for NK-mediated anti-leukemic activity. Thus, the majority of our cases represented KIR2DS1-positive donors with educated NK cells, i.e. those that encountered their appropriate ligand and became unresponsive with respect to alloreactivity. These HLA-C2 unresponsive cells may however have a capability to alleviate autoimmune-like processes such as cGVHD via immunomodulation as described [9].

Interestingly, in our cohort, the strongest differentiating attribute for the favorable effect of rC2+d2DS1 was MAC compared to RIC (Fig 2). A possible explanation may be the well-known fact that, the extended tissue damage, the exposition of new antigens and antigen modification associated with MAC are all strong triggers for alloimmune reactions characteristic to aGVHD and cGVHD [29]. Alternatively, the strong depletion of recipient T cells as observed after MAC but not after RIC may be a permissive factor for donor NK cells to exert their beneficial effect [30].

Upon the dichotomous subgrouping of the entire cohort of 314 patients by age, the combined presence of rC2+d2DS1 showed a favorable outcome only among younger patients (aged below the median). The more prominent role of genetic modifying factors in younger cohorts is well-known compared to the continuously increasing significance of acquired factors in parallel with aging.

Using a comprehensive array of statistical approaches, we observed a clear shift from an effect on relapse rate to substantial improvements in TRM-incidence in the presence of an aKIR and its ligand, HLA-C2 suggesting a beneficial effect of an “educated KIR-ligand constellation”. We assume an “educated” status of KIR2DS1 in all donors with simultaneously positive genotyping results for dDS1 and its cognate ligand, HLA-C2 on the bases of observed mandatory cell surface receptor expression in the presence of the appropriate genes [7].

Behind the observed trend in improvement in EFS in the combined presence of rC2 and dDS1n (Fig 4A) an unequivocal improvement in cumulative TRM-incidence (Fig 4B) and in TRM-incidence rate (Table 4) were found. Our multivariate competing risk models (Table 3) confirmed the lack of effect on relapse (besides the well-known, significant adverse effect of chemo-resistant disease). Our observation that, CMV-reactivation was associated with significantly less relapse was previously described in single center cohorts [31] however; it could not be confirmed in large registry studies [32]. In addition, these analyzes confirmed our expectation that, aGVHD is similarly unaffected by the rC2+d2DS1 combination (besides the well-known significant effect of donor type). This is also supported by the fact that, on the Kaplan-Meier curves (Figs 1B and 3), the OS differences between the rC2+d2DS1 positive vs. negative subgroups become apparent only after approximately the first year post-transplant.

However, we were unable to clearly identify the precise mechanism of the above improvement in TRM-incidence since neither the frequency of cGVHD nor that of infection differed significantly between the two subgroups of TRM-affected patients. Unfortunately, due to insufficient data collection, we were unable to extend these latter analyzes to cGVHD cumulative incidence comparisons. Such a potential cGVHD-modification effect of the simultaneous presence of KIR2DS1 and HLA-C2 was recently demonstrated in a small series of allo-HSCT-treated AML-patients [33]. Potential mechanisms may include regulatory effects exerted by “educated” NK-cells (mediated partly by cell-cell contact or by secreted cytokines e.g. IL-10, interferon-gamma) on immune cells responsible for the initiation and progression of GvHD notably cytotoxic T lymphocytes and/or dendritic cells [34]. An additional potential candidate may be a non-cytotoxic subpopulation of innate lymphoid cells which was shown to participate in the prevention of intestinal GVHD via the production of IL-22 [35]. An overall result of the above immune modulation could be a decreased incidence or milder progression of late transplant complications such as cGVHD that strongly resembles to autoimmune diseases. Such a beneficial modulatory effect has already been documented in the simultaneous presence of KIR2DS1 and HLA-C2 in pregnancy [36] and in multiple sclerosis [37]. Better infection control could be another mechanism behind the observed decreased TRM. In this regard, aKIR molecules in the context of HLA-C2 have been shown to be associated with reduced risk of non-relapse mortality in the haploidentical setting by an enhanced response to infectious agents [38].

Interestingly, among cord blood transplants, HLA-C2 homozygosity conferred higher relapse rate and worse OS when considered alone while in combination with donor KIR genotype, C2/C2 recipients benefited from KIR2DS1 carrier donors [39]. Our observations are in line with these indicating the complexity and variability of the different allo-HSCT cohorts. In another large retrospective registry-analyses of unrelated TX with MAC (60%) and RIC (40%), HLA-C2 homozygous recipients were found to have overall adverse outcomes compared to HLA-C1 positive patients and, the presence of donor KIRDS1-positivity within the HLA-C2 homozygous subgroup was associated with increased treatment related mortality [40]. Our results suggest an opposite trend further emphasizing the need for further studies.

In summary, we identified a genetic combination between recipients and donors of allo-HSCT which may be an independent favorable factor of OS. The rC2+d2DS1 attributable beneficial effect was exclusively characteristic to MAC-treated and younger patients, to later phases of the post-transplant period and was associated with a decreased incidence of TRM. If confirmed, our observations may influence donor selection protocols.

## Supporting information

S1 Table The anonymized table contains all necessary raw data for all statistical analyses performed for the entire patient cohort (n = 314) including critical patient demographic information, outcome parameters, HLAC1/C2 status and KIR-genotyping results. (XLSX)

## References

[1] Shlomchik WD. Graft-versus-host disease. Nature Reviews. Immunology. 2007;7:340–352. 10.1038/nri2000 17438575

[2] Ferrara JL, Levine JE, Reddy P, Holler E. Graft-versus-host disease. Lancet. 2009;373:1550–1561. 10.1016/S0140-6736(09)60237-3 19282026 PMC2735047

[3] Barrett AJ, Battiwalla M. Relapse after allogeneic stem cell transplantation. Expert Review of Hematology. 2010;3:429–441. 10.1586/ehm.10.32 21083034 PMC3426446

[4] Chien JW, Zhao LP, Storer B, Martin PJ, Boeckh M, Warren EH, et al Improving Hematopoietic Cell Transplant Outcomes in a New Era of Genomic Research. Biology of Blood and Marrow Transplantation. 2009;15:42–45. 10.1016/j.bbmt.2008.11.003 19147077 PMC2946198

[5] Dickinson AM. Non-HLA genetics and predicting outcome in HSCT. International Journal of Immunogenetics. 2008;35:375–380. 10.1111/j.1744-313X.2008.00790.x 18976441

[6] Hansen JA, Chien JW, Warren EH, Zhao LP, Martin PJ. Defining genetic risk for graft-versus-host disease and mortality following allogeneic hematopoietic stem cell transplantation. Current Opinion in Hematology. 2010;17:483–492. 10.1097/MOH.0b013e32833eb770 20827186 PMC3177530

[7] Foley B, Felices M, Cichocki F, Cooley S, Verneris MR, Miller JS. The biology of NK-cells and their receptors affects clinical outcomes after hematopoietic cell transplantation (HCT). Immunological Reviews. 2014;258:45–63. 10.1111/imr.12157 24517425 PMC3927144

[8] Stringaris K, Barrett AJ. The importance of natural killer cell killer immunoglobulin-like receptor-mismatch in transplant outcomes. Curr Opin Hematol. 2017;24:489–495. 10.1097/MOH.0000000000000384 28817402

[9] Boudreau JE, Hsu KC. Natural killer cell education in human health and disease. Curr Opin Immunol. 2018;50:102–111. 10.1016/j.coi.2017.11.003 29413815 PMC5958620

[10] Sivori S, Carlomagno S, Falco M, Romeo E, Moretta L, Moretta A. Natural killer cells expressing the KIR2DS1-activating receptor efficiently kill T-cell blasts and dendritic cells: implications in haploidentical HSCT. Blood. 2011;117:4284–4292. 10.1182/blood-2010-10-316125 21355085

[11] Kannan GS, Aquino-Lopez A, Lee DA. Natural killer cells in malignant hematology: A primer for the non-immunologist. Blood Rev. 2017;31:1–10. 10.1016/j.blre.2016.08.007 27665023

[12] Babor F, Fischer JC, Uhrberg M. The role of KIR genes and ligands in leukemia surveillance. Frontiers in Immunology. 2013;4:27 10.3389/fimmu.2013.00027 23404428 PMC3566379

[13] Symons HJ, Fuchs EJ. Hematopoietic SCT from partially HLA-mismatched (HLA-haploidentical) related donors. Bone Marrow Transplant. 2008;42:365–77. 10.1038/bmt.2008.215 18679375 PMC2699592

[14] Venstrom JM, Pittari G, Gooley TA, Chewning JH, Spellman S, Haagenson M, et al HLA-C–Dependent Prevention of Leukemia Relapse by Donor ActivatingKIR2DS1. New England Journal of Medicine. 2012;367:805–816. 10.1056/NEJMoa1200503 22931314 PMC3767478

[15] Nowak J, Kościńska K, Mika-Witkowska R, Rogatko-Koroś M, Mizia S, Jaskuła E, et al Role of Donor Activating KIR–HLA Ligand–Mediated NK-cell Education Status in Control of Malignancy in Hematopoietic Cell Transplant Recipients. Biology of Blood and Marrow Transplantation. 2015;21:829–839. 10.1016/j.bbmt.2015.01.018 25617806

[16] Hsu KC, Keever-Taylor CA, Wilton A, Pinto C, Heller G, Arkun K, et al Improved outcome in HLA-identical sibling hematopoietic stem-cell transplantation for acute myelogenous leukemia predicted by KIR and HLA genotypes. Blood. 2005;105:4878–4884. 10.1182/blood-2004-12-4825 15731175 PMC1894998

[17] Nowak J, Kościńska K, Mika-Witkowska R, Rogatko-Koroś M, Mizia S, Jaskuła E, et al Donor NK-cell licensing in control of malignancy in hematopoietic stem cell transplant recipients. American Journal of Hematology. 2014;89:E176–E183. 10.1002/ajh.23802 25044365

[18] Cooley S, Weisdorf DJ, Guethlein LA, Klein JP, Wang T, Le CT, et al Donor selection for natural killer cell receptor genes leads to superior survival after unrelated transplantation for acute myelogenous leukemia. Blood. 2010;116:2411–2419. 10.1182/blood-2010-05-283051 20581313 PMC2953880

[19] Oevermann L, Michaelis SU, Mezger M, Lang P, Toporski J, Bertaina A, et al KIR B haplotype donors confer a reduced risk for relapse after haploidentical transplantation in children with ALL. Blood. 2014;124:2744–7. 10.1182/blood-2014-03-565069 25115891 PMC4208288

[20] Bachanova V, Weisdorf DJ, Wang T, Marsh SGE, Trachtenberg E, Haagenson MD, et al Donor KIR B Genotype Improves Progression-Free Survival of Non-Hodgkin Lymphoma Patients Receiving Unrelated Donor Transplantation. Biology of Blood and Marrow Transplantation. 2016;22:1602–1607. 10.1016/j.bbmt.2016.05.016 27220262 PMC4981536

[21] Hosokai R, Masuko M, Shibasaki Y, Saitoh A, Furukawa T, Imai C. Donor Killer Immunoglobulin-Like Receptor Haplotype B/x Induces Severe Acute Graft-versus-Host Disease in the Presence of Human Leukocyte Antigen Mismatch in T Cell–Replete Hematopoietic Cell Transplantation. Biology of Blood and Marrow Transplantation. 2017;23:606–611. 10.1016/j.bbmt.2016.12.638 28042021

[22] Przepiorka D, Weisdorf D, Martin P, Klingemann HG, Beatty P, Hows J, et al 1994 Consensus Conference on Acute GVHD Grading. Bone Marrow Transplant. 1995;15:825–828. 7581076

[23] Shulman HM, Sullivan KM, Weiden PL, McDonald GB, Striker GE, Sale GE, et al Chronic graft-versus-host syndrome in man. A long-term clinicopathologic study of 20 Seattle patients. Am J Med. 1980;69:204–17. 6996481 10.1016/0002-9343(80)90380-0

[24] Abalos AT, Eggers R, Hogan M, Nielson CM, Giuliano AR, Harris RB, et al Design and validation of a multiplex specific primer-directed polymerase chain reaction assay for killer-cell immunoglobulin-like receptor genetic profiling. Tissue Antigens. 2011;77:143–148. 10.1111/j.1399-0039.2010.01588.x 21214526

[25] Kanda Y. Investigation of the freely available easy-to-use software 'EZR' for medical statistics. Bone Marrow Transplant. 2013;48:452–458. 10.1038/bmt.2012.244 23208313 PMC3590441

[26] Delgado J, Pereira A, Villamor N, López-Guillermo A, Rozman C. Survival analysis in hematologic malignancies: recommendations for clinicians. Haematologica. 2014;99:1410–1420. 10.3324/haematol.2013.100784 25176982 PMC4562529

[27] Austin PC, Lee DS, Fine JP. Introduction to the Analysis of Survival Data in the Presence of Competing Risks. Circulation. 2016;133:601–609. 10.1161/CIRCULATIONAHA.115.017719 26858290 PMC4741409

[28] Sebestyén A, Mester S, Vokó Z, Gajdácsi J, Cserháti P, Speer G, et al Wintertime surgery increases the risk of conversion to hip arthroplasty after internal fixation of femoral neck fracture. Osteoporos Int. 2015;26:1109–1117. 10.1007/s00198-014-2966-0 25472855

[29] Brennan TV, Rendell VR, Yang Y. Innate immune activation by tissue injury and cell death in the setting of hematopoietic stem cell transplantation. Front Immunol. 2015;6:101 10.3389/fimmu.2015.00101 25852683 PMC4360715

[30] Jagasia M, Arora M, Flowers ME, Chao NJ, McCarthy PL, Cutler CS et al Risk factors for acute GVHD and survival after hematopoietic cell transplantation. Blood. 2012;119:296–307. 10.1182/blood-2011-06-364265 22010102 PMC3251233

[31] Green ML, Leisenring WM, Xie H, Walter RB, Mielcarek M, Sandmaier BM, et al CMV reactivation after allogeneic HCT and relapse risk: evidence for early protection in acute myeloid leukemia. Blood. 2013;122:1316–24. 10.1182/blood-2013-02-487074 23744585 PMC3744995

[32] Teira P, Battiwalla M, Ramanathan M, Barrett AJ, Ahn KW, Chen M, et al Early cytomegalovirus reactivation remains associated with increased transplant-related mortality in the current era: a CIBMTR analysis. Blood. 2016;127:2427–38. 10.1182/blood-2015-11-679639 26884374 PMC4874224

[33] Gaafar A, Sheereen A, Almohareb F, Eldali A, Chaudhri N, Mohamed SY, et al Prognostic role of KIR genes and HLA-C after hematopoietic stem cell transplantation in a patient cohort with acute myeloid leukemia from a consanguineous community. Bone Marrow Transplant. 2018;53:1170–1179. 10.1038/s41409-018-0123-7 29549293

[34] Crouse J, Xu HC, Lang PA, Oxenius A. NK-cells regulating T cell responses: mechanisms and outcome. Trends in Immunology. 2015;36:49–58. 10.1016/j.it.2014.11.001 25432489

[35] Konya V, Mjösberg J. Innate Lymphoid Cells in Graft-Versus-Host Disease. American Journal of Transplantation. 2015;15:2795–2801. 10.1111/ajt.13394 26228632 PMC4973689

[36] Hiby SE, Apps R, Sharkey AM, Farrell LE, Gardner L, Mulder A, et al Maternal activating KIRs protect against human reproductive failure mediated by fetal HLA-C2. J Clin Invest. 2010;120:4102–10. 10.1172/JCI43998 20972337 PMC2964995

[37] Fusco C, Guerini FR, Nocera G, Ventrella G, Caputo D, Valentino MA, et al KIRs and their HLA ligands in remitting-relapsing multiple sclerosis. J Neuroimmunol. 2010;229:232–7. 10.1016/j.jneuroim.2010.08.004 20826009

[38] Mancusi A, Ruggeri L, Urbani E, Pierini A, Massei MS, Carotti A, et al Haploidentical hematopoietic transplantation from KIR ligand-mismatched donors with activating KIRs reduces nonrelapse mortality. Blood. 2015;125:3173–3182. 10.1182/blood-2014-09-599993 25769621

[39] Sekine T, Marin D, Cao K, Li L, Mehta P, Shaim H, et al Specific combinations of donor and recipient KIR-HLA genotypes predict for large differences in outcome after cord blood transplantation. Blood. 2016;128:297–312. 10.1182/blood-2016-03-706317 27247137 PMC4946205

[40] Neuchel C, Fürst D, Niederwieser D, Bunjes D, Tsamadou C, Wulf G, et al Impact of Donor Activating KIR Genes on HSCT Outcome in C1-Ligand Negative Myeloid Disease Patients Transplanted with Unrelated Donors-A Retrospective Study. PLoS One. 2017;12:e0169512 10.1371/journal.pone.0169512 28107369 PMC5249182

